# A transdiagnostic approach to neurodiversity in a representative population sample: The N+ 4 model

**DOI:** 10.1002/jcv2.12219

**Published:** 2024-02-01

**Authors:** Ian A. Apperly, Robert Lee, Sanne W. van der Kleij, Rory T. Devine

**Affiliations:** ^1^ Centre for Developmental Science School of Psychology University of Birmingham Birmingham UK

**Keywords:** ADHD, autism, broader phenotype, dyslexia, neurodiversity, transdiagnostic

## Abstract

**Background:**

The concept of neurodiversity draws upon scientific research, and lessons from practice and lived experience to suggest new ways of thinking about neurodevelopmental conditions. Among the formative observations are that characteristics associated with neurodevelopmental conditions are part of a “broader phenotype” of variation across the whole population, and that there appear to be “transdiagnostic” similarities as well as differences in these characteristics. These observations raise important questions that have implications for understanding diversity in neurodevelopmental conditions and in neurocognitive phenotypes across the whole population.

**Method:**

The present work examines broader phenotypes using seven widely used self‐report assessments of traits associated with autism, ADHD, dyslexia, Developmental Coordination Disorder/dyspraxia, tic disorders/Tourette's, cortical hyperexcitability associated with subclinical epilepsy, and sensory sensitivities. A representative sample of 995 adults (aged 17–77) in the UK completed self‐report measures of neurodiversity, wellbeing, generalized anxiety, and depression, and cognitive abilities (nonverbal intelligence and executive functioning).

**Results:**

We used confirmatory factor analysis to test whether variation and covariation was better characterized (1) by traditional diagnostic labels, or (2) transdiagnostically according to similarities in functions, behaviours, or phenomena. Results indicated that neurodiversity characteristics were best explained using a bifactor model with one general “N” factor and four condition‐specific factors.

**Conclusion:**

This was the largest examination to date of the factor structure of broader phenotypes relevant to neurodevelopmental conditions. It provides critical benchmark data, and a framework approach for asking systematic questions about the structure of neurocognitive diversities seen in the whole population and in people with one or more diagnoses.

## Graphical abstract

For self‐report broader phenotype measures for six neurodevelopmental conditions a single “N‐factor” accounted for the most overall variance on all measures, whilst four further factors accounted for additional unique variance in traits associated with autism, ADHD, cortical hyperexcitability, and dyslexia/DCD. This N + 4 factor structure suggests that broader phenotype scales measure both general “neurodiversity” and traits that are more condition‐specific. Distinguishing these sources of variance may be helpful for understanding the complex patterns of cognition, behaviour, and experience associated with neurodiversity.

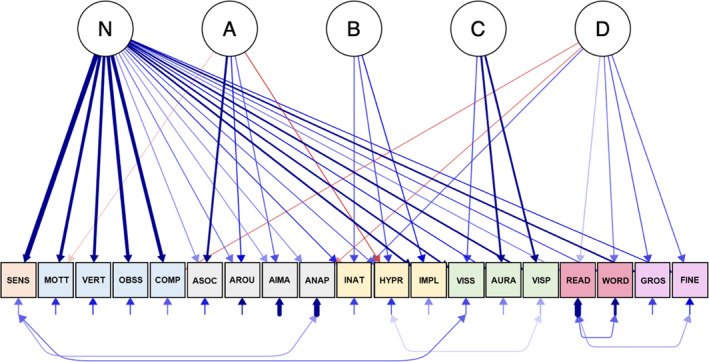

**Key Points**

- **Known:** Neurodevelopmental conditions show considerable phenotypic variability, and high rates of co‐diagnosis. Cognitive and behavioural characteristics associated with these conditions show “broader phenotypes” across the whole population.
- **New:** We employed self‐report broader phenotype measures for six neurodevelopmental conditions. Bifactor modelling revealed that a single N factor accounted for variance on all measures, and the largest proportion of variance overall. Four further factors accounted for additional unique variance in traits often associated with autism, ADHD, cortical hyperexcitability, and dyslexia/Developmental Coordination Disorder (DCD) respectively.
- **Relevance:** The N + 4 factor structure suggests that broader phenotype scales measure both general “neurodiversity”, and variability in traits that are more condition specific. Distinguishing these sources of variance may be valuable for understanding the highly variable experiences and needs of people with a neurodevelopmental diagnosis.

## INTRODUCTION

The concept of neurodiversity draws upon lessons from lived experience, clinical and educational practice, and scientific research to suggest new ways of thinking about neurodevelopmental conditions (e.g., Fletcher‐Watson, 2022). One formative set of observations concerns “complexity”.^1^ There are many differences between individuals diagnosed with a given neurodevelopmental condition, and there also appear to be “transdiagnostic” similarities, and co‐occurrence of neurodevelopmental conditions is common (e.g., Astle et al., 2022). Another formative observation is that characteristics associated with neurodevelopmental conditions are part of a “broader phenotype”^2^ of variation across the whole population. These observations have implications for how we conceptualise and study diversity in neurocognitive phenotypes. For example, such observations raise questions about whether the standard diagnostic categories give the best account of the complex patterns of variation and highlight the relevance of examining patterns of variance in the whole population. In the present work we pursue these questions by examining the factor structure of individual differences in traits associated with six neurodevelopmental conditions in a representative sample of 995 adults from the United Kingdom.

### Neurodiversity concepts and terminology

The term “neurodiversity” originated in the autistic advocacy movement that challenged mainstream medical models by conceptualising autism in terms of neurocognitive differences, rather than pathologies (Blume, 1998; Singer, 1999). The term's scope has since broadened to include other neurodevelopmental conditions (e.g., Fletcher‐Watson, 2022), and its meanings have become diverse and contested (e.g., Dwyer, 2022). However, a common thread remains that neurodevelopmental conditions are part of a bigger picture of variation in neurocognitive phenotypes across the whole population. This has generated related terminology, whereby people whose neurocognitive profiles are more frequent in the population are described as “neurotypical”, while those with less frequently observed profiles (and who might meet diagnostic criteria for a neurodevelopmental condition) are described as “neurodivergent” (e.g., Pellicano & den Houting, 2022).

Neurodevelopmental conditions are often distinguished from psychiatric conditions, which are thought to show cycles of remittance and relapse that contrast with the temporal stability and early childhood emergence of neurodevelopmental conditions (e.g., Thapar et al., 2017). The security of this distinction is debatable. Arguments against the distinction include the observations that some “psychiatric” conditions—such as schizophrenia—show patterns of developmental emergence that resemble “neurodevelopmental” conditions (e.g. Birnbaum & Weinberger, 2017; Rapoport et al., 2012), that there is considerable overlap in the genetic predisposition towards both schizophrenia and other neurodevelopmental conditions such as autism and ADHD (Owen & O'Donovan, 2017), and that many neurodevelopmental and psychiatric conditions co‐occur at higher levels than expected by chance suggesting they may have causes in common (e.g. Addicoat et al., 2020; Eberhard et al., 2022; England‐Mason, 2020). The latter argument has been countered by the suggestion that the demands of living with a neurodevelopmental condition in a largely neurotypical world may itself be a cause of at least some co‐occurrent psychiatric conditions (Alexander‐Passe, 2015; Cage et al., 2018; Cage & Troxell‐Whitman, 2019; Dwyer, 2022; Gallant & Good, 2023; Kiraz & Sertçelik, 2021; Mantzalas et al., 2022; Pryke‐Hobbes et al., 2023; Reindal, 2008). In the present work we follow the common practice of distinguishing neurodevelopmental and psychiatric conditions, and we model our data on self‐rated depression, anxiety, and mental wellbeing separately from neurodiversity traits. However, our study was not designed to test these working assumptions and cannot provide evidence for or against them.

### Neurodevelopmental conditions as complex phenotypes

It is increasingly recognised that neurodevelopmental conditions are “complex”, and that this complexity matters for recognising and providing for individual needs, and for conceptualising research (Embracing Complexity, 2021). The phenotypes of people with a particular diagnosis can be highly variable. For example, the DSM‐V‐TR criteria for autism include: stereotyped or repetitive motor movements, use of objects, or speech; insistence on sameness, inflexible adherence to routines, or ritualized patterns of verbal or nonverbal behaviour; highly restricted, fixated interests that are abnormal in intensity or focus; hyper‐reactivity or hypo‐reactivity to sensory input or unusual interest in sensory aspects of the environment (APA, 2022, pp. 50–59). Diagnosis requires the presence of two (or more) of these criteria in any combination. This yields 10 unique combinations, without taking account of the distinctive ways in which individuals might meet any criterion. This complexity becomes even more pronounced in the case of phenotypes for ADHD (APA, 2022, pp. 59–66); both the DSM‐V‐TR criteria for inattentive ADHD and the criteria for hyperactive‐impulsive ADHD each require six or more symptoms to be present out of a list of nine potential symptoms, or five out of the nine for individuals aged 17 or older—for each separate diagnosis in adults this yields 84 unique combinations (126 combinations for individuals being diagnosed when aged 17 or older), or 7056 unique combinations in the case of a diagnosis of combined inattentive and hyperactive‐impulsive ADHD (15,876 combinations for people undergoing the diagnostic process when aged 17 or older). Whilst a diagnosis of dyslexia cannot be met by such varied combinations of symptoms, it is located within the DSM‐V‐TR under the broader category of ‘Specific Learning Disorder’ and any one of the following four symptoms is sufficient (along with other mandatory criteria) for a diagnosis: inaccurate or slow and effortful word reading; difficulty understanding the meaning of what is read; difficulties with spelling; or difficulties with written expression (APA, 2022, pp. 66–74).

It is also very common for a person with one diagnosis to meet the criteria for one or more other diagnoses. For example, meta‐analysis indicates that the prevalence of ADHD among autistic people is approximately 40% (Rong et al., 2021), 14% of autistic people may have dyslexia (Hofvander et al., 2009), and 5%–46% experience epileptic seizures (Ghacibeh & Fields, 2015). Approximately 45% of people with ADHD may also have a specific learning difficulty (DuPaul et al., 2013), 47% may have challenges with motor skills (Farran et al., 2020), 13% may meet criteria for an autism diagnosis (Zablotsky et al., 2020), and 1.2% may have Tourette's (Danielson et al., 2018). Over 50% of children with dyslexia may also have motor difficulties associated with DCD (Chaix et al., 2007), and over 50% also met the criteria for ADHD (Hirschtritt et al., 2015), and the co‐occurrence of ADHD amongst people with epilepsy is between 28% and 70% (Berl et al., 2015).

Finally, while diagnostic categories emphasise “core” features that are distinctive of a particular condition, other “non‐core” features may be equally important or sometimes more important contributors to people's experience and needs. Such observations put pressure on traditional diagnostic categories and have led some to question the validity of categorical diagnoses and the utility of these diagnoses as selection criteria for research (e.g., Astle et al., 2022). Others have argued that diagnostic categories retain utility, but that they should be combined with new approaches that do justice to complexity that is not well‐captured by a categorical approach (e.g., Dwyer, 2022; Happé & Frith, 2020). In what follows we lay groundwork for one such approach to this challenge.

### Broader phenotypes

While the practice of diagnosis and the tradition of case‐control studies emphasises categorical distinctions between people “with” and “without” a particular condition, there is also evidence that many people with neurodevelopmental conditions tend towards the extremes of a phenotypic “spectrum” that runs across the whole population (e.g., Baron‐Cohen et al., 2001; Conners et al., 2003; Fong et al., 2019; Gaffney et al., 1994; Happé & Frith, 2020; Kirby et al., 2010; Snowling et al., 2012). There are several ways in which this “broader phenotype” approach fits well with concepts of neurodiversity. The approach captures “diversity” in terms of continuous traits rather than categorical distinctions. Broader phenotype measures often include sub‐scales to measure different phenomena to reflect the idea that different people can have distinct profiles of traits associated with that condition.

Moreover, there is no logical or methodological barrier to profiling an individual on multiple broader phenotype measures, reflecting the possibility that one person could show high levels of traits for one, many, or none of the conditions. However, this line of thinking leads to a challenge of spiralling complexity, given the many ways in which dimensions of diversity could be combined. In the present work this challenge is addressed by using factor analysis to examine the underlying sources of covariance across multiple measures.

### Sampling from the whole population

The broader phenotype perspective suggests that variance in traits related to neurodevelopmental conditions can be observed across the whole population. This transdiagnostic approach to recruitment captures those who experience differences that do not meet standard clinical cut‐offs and provides an opportunity to investigate naturally occurring variation and covariation in neurocognitive phenotypes (e.g., Astle et al., 2022). While this approach does not guarantee that patterns seen in the whole population will be the same for people with a clinical diagnosis, a sample representing a whole population enables us to establish a foundation for future work that examines the consistency of the factor structure in different groups within the population. It can also be motivated independently, as a distinctive perspective on human neurocognitive diversity.

### Transdiagnostic approaches to psychiatry and neurodevelopmental conditions

As for neurodevelopmental conditions there are high levels of co‐occurrence of different psychiatric conditions, and there is considerable variability between individuals with the same diagnosis. This has led researchers to question common psychiatric diagnostic categories (e.g., Robbins et al., 2012). One approach to this challenge uses factor analysis to examine the statistical structure of symptoms across large samples of people with diverse psychiatric diagnoses (e.g., Caspi & Moffitt, 2018). Moreover, given the high rates of co‐occurrence, such work has sometimes included assessments of characteristics related to neurodevelopmental conditions (e.g., Bloemen et al., 2018; Nordhof et al., 2015). This work suggests that some variation in symptoms is explained by specific diagnostic factors, and some by super‐ordinate dimensions of “externalising” and “internalising” (Caspi & Moffitt, 2018). However, the largest portion of variation is accounted for by a single “p‐factor” that cuts across all diagnoses (e.g., Caspi et al., 2014). The p‐factor provides a way of conceptualising commonalities in the nature and severity of symptoms, “liability” for clinical diagnosis, and high rates of diagnosis with multiple conditions. In the present work we adopted a factor analytic approach to investigate the sources of overlap between neurodiversity characteristics, while remaining agnostic about the relationship between neurodevelopmental and psychiatric conditions.

### Summary of aims

**FIGURE 1:**
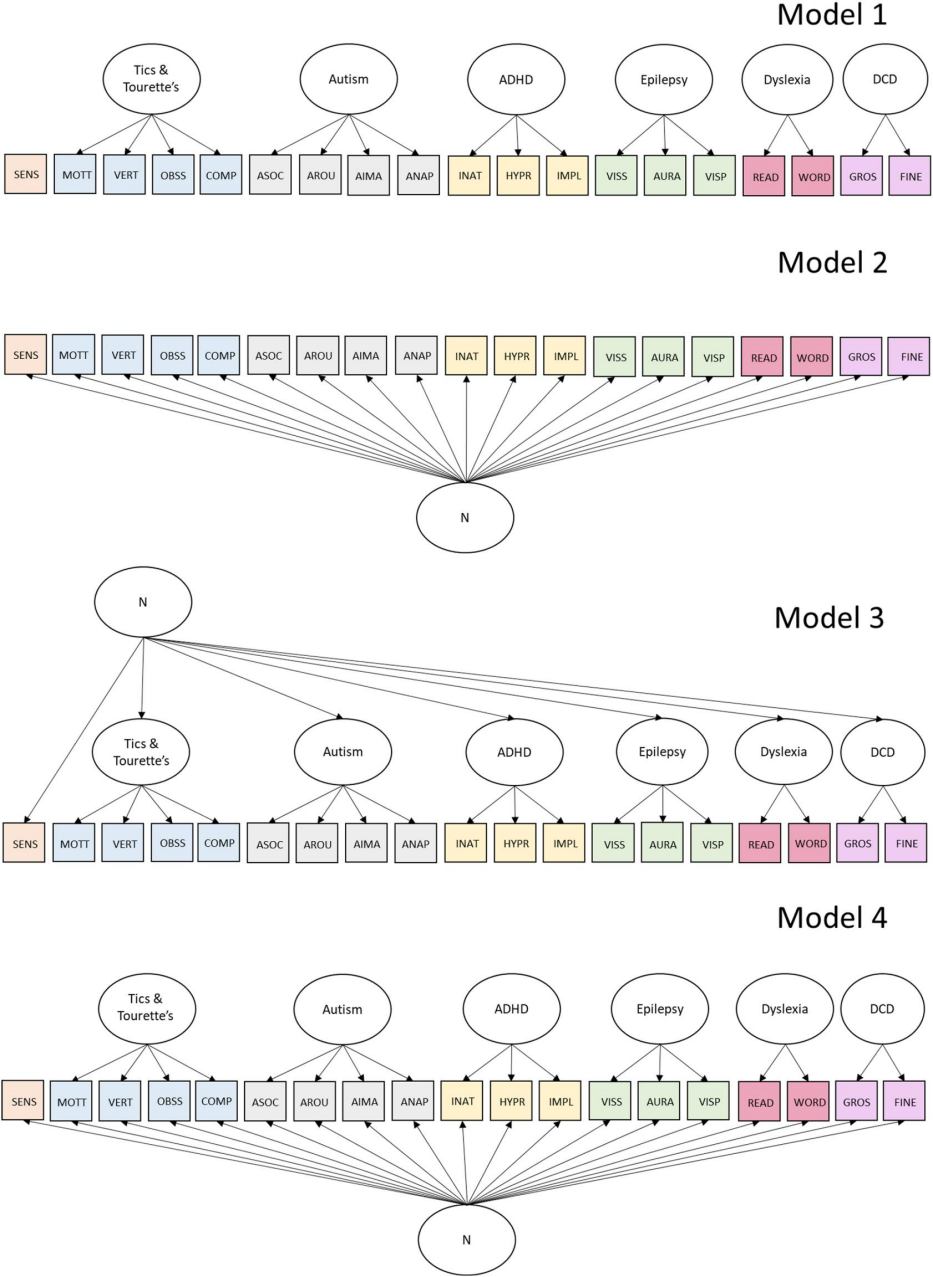
Theory‐Driven Measurement Models modelling the underlying structure of individual differences in neurodiversity traits. SENS = GSQ Sensory Sensitivities. MOTT = MOVES Motor Tics. VERT = MOVES Verbal Tics. OBSS = MOVES Obsessions. COMP = MOVES Compulsions. ASOC = AQ Social Skills Difficulties. AROU = AQ Preference for Routines. AIMA = AQ Imagination. ANAP = AQ Numbers and Patterns. INAT = CAARS Inattentiveness. HYPR = CAARS Hyperactivity. IMPL = CAARS Impulsivity. VISS = CHI‐II Heightened Visual Sensitivity. AURA = CHI‐II Aura‐Like Hallucinatory Experiences. VISP = CHI‐II Distorted Visual Perception. READ = ARQ Reading Difficulties. WORD = ARQ Word Finding Difficulties. GROS = ADC‐R Gross Motor Skills. FINE = ADC‐R Fine Motor Skills.

We investigated the latent factor structure of neurodiversity by examining individual differences in characteristics associated with autism, ADHD, dyslexia, dyspraxia, tic disorders/Tourette's, cortical hyperexcitability associated with epilepsy, and sensory sensitivities. These were chosen because the associated conditions are among the most frequent neurodevelopmental conditions (Cleaton & Kirby, 2018; Francés et al., 2022; Straub et al., 2022; Zablotsky et al., 2019) and had broader phenotype scales with two or more subscales. The exception to this principle was the scale of sensory sensitivities, which is a single scale. This was included because sensory sensitivities are widely reported in different neurodevelopmental conditions but do not routinely feature in current broader phenotype scales (Ward, 2019). A ‘status quo’ model would suggest that neurodiversity characteristics are driven by separable (but sometimes correlated) condition‐specific phenotypes. An alternative view is that neurodiversity characteristics co‐occur because they can be explained by a common factor (or common factors) that transcends traditional condition categories. Our first aim was to use theory‐driven and data‐driven latent variable modelling to examine these alternative underlying structures of individual differences in neurodiversity traits (Figure 1).

Our second aim was to test whether the best model of the data was redundant with other measures (such as general cognitive ability), whether components of the model showed selectivity (showing distinctive patterns of correlation with other measures), and whether the model showed relevance to people's everyday wellbeing. Given evidence of high levels of depression and anxiety among many people with diagnosed neurodevelopmental conditions (e.g., Howlin & Magiati, 2017; Katzman et al., 2017) we assessed relevance by examining relationships with participants' self‐rated depression, anxiety, and general wellbeing. Our research questions and analysis plan were pre‐registered on the Open Science Framework prior to data collection (https://osf.io/rtsjy).

## MATERIALS AND METHOD

### Participants

A target sample of 1000 participants was determined a priori using Monte Carlo simulations in Mplus (see Supporting Information: Power Analysis for further detail). Eighteen participants (7%) were excluded due to potentially confounding neurological, visual, and hearing conditions. Of the final sample (*N* = 995), 503 identified as women, 484 as men, 4 as non‐binary/other; age ranged from 17 to 77 (*M* = 44.55, SD = 15.64). 78.3% of participants were White British, 6.36% were Asian British, 3.43% were Black British, 2.42% were of mixed race, and 8.27% were of other ancestry. Participants used the MacArthur Scale of Subjective Social Status—Adult Version (Adler et al., 2000) to self‐assess their relative socio‐economic status (*M* = 5.28, SD = 1.514, Range 1–9). More detailed demographic information about the sample can be found on our Open Science Framework project page: https://osf.io/cmywb/?view_only=7b11380503464c6e89ddf622ab8cbd4d.

### Procedure

Approval was obtained from the University of Birmingham STEM Ethics committee, (ERN_22–1192). An online survey was constructed from self‐report scales designed to measure traits related to common neurodevelopmental conditions. Participants were recruited online via Prolific (www.prolific.co) [date accessed 08.05.2023], using their nationally representative sampling criteria for UK residents (ethnicity by gender for each age category, Prolific, 2023) and completed a survey on Qualtrics with questions grouped according to measure.

### Measures

The self‐report scales were selected to be reliable for adults, to have subscales corresponding to key trait domains of each condition, and to be sensitive to individual differences in the “broader phenotype” for each trait in non‐clinical samples. The scales were presented in the order listed in Table 1.

**TABLE 1:** Scales completed by participants.

Name of scale	Likert scale	Subscales used	Modifications and notes
Autism quotient (AQ, Baron‐Cohen et al., 2001)	4‐Point: Definitely agree [1], slightly agree [2], slightly disagree [3], definitely disagree [4] (some items reverse‐coded)	Hoekstra et al. (2011): Social skills [ASOC] (4 of 8 items), Routine [AROU] (3 of 4 items), imagination [AIMA] (4 of 8 items), numbers and patterns [ANAP] (5 of 5 items)	See footnote^a^
Adult reading questionnaire (ARQ, Snowling et al., 2012)	Questions 1 & 2: 3‐Point (yes [0], no [1], don't know/unsure [0.5→1]). Question 3: 4‐Point (good [0], average [1], poor [2], very poor [3]), questions 4–8: 5‐Point (never [0], rarely [1], sometimes [2], frequently [3], always [4])	Reading [READ] (5 questions), Word finding [WORD] (3 questions)	All questions whose scores did not load onto the 2 subscales were removed. ‘Don't know/unsure’ [0.5] was coded onto ‘No’ [1] (questions 1 and 2)
Conners adult ADHD rating scale (CAARS, Conners et al., 2003)	4‐Point: Not true at all (Never, Seldom) [0], just a little true (Occasionally) [1], Pretty much true (often, quite a bit) [2], very much true (very often, very frequently) [3]	Inattention/Memory problems [INAT] (12 questions), Hyperactivity/Restlessness [HYPR] (12 questions), Impulsivity/Emotional Lability [IMPL] (12 questions)	Problems with self‐ concept subscale removed (6 questions)
Glasgow sensory questionnaire (GSQ, Robertson & Simmons, 2013)	5‐Point: Never [0], rarely [1], sometimes [2], often [3], Always [4]	Total scale used [SENS] (42 questions, single factor scale, 21 hypersensitivity, 21 hyposensitivity)	None
Motor tic, obsessions and compulsions, vocal tic evaluation survey (MOVES, Gaffney et al., 1994)	4‐Point scale: Never [0], sometimes [1], often [2], always [3]	Motor tics [MOTT] (4 questions), vocal tics [VERT] (4 questions), obsessions [OBSS] (4 questions), compulsions [COMP] (4 questions)	4 questions on ‘associated symptoms’ removed: Echolalia, echopraxia, coprolalia, copropraxia
Revised adult developmental coordination disorders/dyspraxia checklist (ADC‐R, Meachon et al., 2022)	‘Never’ [1], ‘sometimes’ [2], ‘frequently’ [3] or ‘always’ [4]	Gross motor difficulties [GROS] (16 questions), Fine motor difficulties [FINE] (10 questions)	Executive functions subscale removed (11 questions)
Cortical hyperexcitability index—II (CHi‐II, Fong et al., 2019)	Two 7‐point scales for each question: Frequency (0 = never to 6 = all the time) and intensity (0 = not at all to 6 = extremely intense)	Heightened visual sensitivity and discomfort [VISS] (11 questions), aura‐like Hallucinatory experiences [AURA], (9 questions), distorted visual perception VISP] (6 questions)	Version of scale used where all questions load onto a distinct factor
Abbreviated Raven's progressive matrices task (RPMT, Bilker et al., 2012)	Multiple choice, 1 correct answer per question. Questions 1–2 had 6 options, questions 3–9 had 8 options	Total scale used (9 questions from original 60 RMPT questions: Set A as produced by Bilker et al., 2012) to produce a single score	Used as a proxy measure for non‐verbal ability
Warwick– Edinburgh Mental well‐being scale (WEMWBS, Tennant et al., 2007)	5‐Point measuring frequency over the past 2 weeks: None of the time [1], rarely [2], some of the time [3], often [4], all of the time [5]	Total scale used (14 questions)	Higher scores on WEMWBS indicate higher levels of mental wellbeing.
Generalized anxiety disorder assessment (GAD‐7, Spitzer et al., 2006)	4‐Point measuring frequency over the past 2 weeks: Not at all [1], several days [2], more than half the days [3], nearly every day [4]	Total scale used (7 questions)	Scores from WEMWBS (reverse‐coded), GAD‐7 and PHQ‐9 were summed into a combination scale, which indicated self‐assessed challenges with mental health and wellbeing
Patient health questionnaire (PHQ‐9, Kroenke et al., 2001)	4‐Point measuring frequency over the past 2 weeks: Not at all [1], several days [2], more than half the days [3], nearly every day [4]	Total scale used (9 questions)	
Executive function index (EFI, Spinella, 2005)	5‐Point Likert scale: Not at all [1], [2], somewhat [3], [4], very much [5] (12 items reverse‐coded)	Empathy (6 questions), strategic planning (7 questions), organization (5 questions), impulse control (5 questions)	Motivational drive subscale cut (4 questions).
			Used as a proxy for EF skills (higher scores indicate greater EF skills)

^a^ A 28‐item version of the Autism Quotient (AQ, Baron‐Cohen et al., 2001) by Zhu et al. (2022) was administered to assess traits related to autism. Participants responded on a 4‐point Likert Scale, with responses summed for each subscale. Preliminary analyses of the subscales indicated that scores measuring social and communication preferences did not correlate with each other as expected (e.g., social anhedonia and [participation in] social discourse conventions, *r* (987) = .04). In a departure from our pre‐registration, we opted for a simpler scoring scheme based on four subscales identified by Hoekstra et al. (2011): Social skills, Routine, Imagination, and Numbers & Patterns.

## RESULTS

### Descriptive statistics

See Table S1 for the descriptive statistics, internal consistencies and correlations for each measure included in the study. Attention‐check items were embedded within each scale (11 in total). If participants failed an attention‐check item by selecting an incorrect response, their data for that scale were treated as missing (*N* = 34 failed at least 1 attention‐check; *N* = 2 failed 2 attention checks, and *N* = 2 failed 3 attention‐checks).

### Latent factor structure of neurodiversity traits/characteristics

As specified in the pre‐registration, we conducted latent variable modelling in Mplus (Muthèn & Muthèn, 2017) using a robust maximum likelihood (MLR) estimator in each of our analyses (Roos & Bauldry, 2022). Missing data were handled using Full Information MLR estimation (Roos & Bauldry, 2022). We evaluated overall model fit using standard criteria: root mean square error of approximation (RMSEA) <0.08, a comparative fit index > 0.90, and Tucker Lewis Index > 0.90 (Brown, 2015). We used modification indices to assess for areas of model strain. To do this, we examined the highest modification index relating to correlated residual terms and respecified the model if the expected parameter change was theoretically interpretable (Roos & Bauldry, 2022). For assessment of measurement models, we randomly allocated our participants into two datasets. The test dataset consisted of 498 participants, *M* Age = 44.66 years, SD = 15.29, 50.8% female. The hold‐out dataset for cross‐validation consisted of 497 participants, *M* Age = 44.44 years, SD = 16.05, 51.7% female. We then re‐combined the two datasets to examine correlates of neurodiversity using latent variable modelling.

We first tested four theory‐driven models (see Table 2). Each of these models provided a poor fit to the data. We inspected modification indices for each of these models and respecified each model to incorporate correlated residual terms. However, these modifications did not lead to acceptable model fit. Next, we adopted a data‐driven approach using exploratory factor analysis with oblique Geomin rotation to estimate a first‐order solution incorporating between 1 and 6 latent factors. We also used exploratory bifactor modelling with orthogonal Geomin rotation to compare competing bifactor solutions with 1 general factor and between 1 and 5 specific factors. Results of these EFAs are shown in Table 1. A five‐factor solution provided the best fitting first‐order model (B5). A bifactor model with one general factor and four specific factors provided the best fitting bifactor model (B9). We tested these two models using confirmatory factor analysis (CFA).

**TABLE 2:** Model fit indices for measurement models.

	Description	***χ*** ^2^	*df*	CFI	TLI	RMSEA	AIC	BIC
Theory‐driven confirmatory factor analysis								
A1	One factor model	1231.25	152	0.67	0.63	0.12	51,687.27	51,927.27
A2	Six correlated factors, one indicator	563.00	132	0.87	0.83	0.08	50,913.39	51,237.61
A3	Second‐order factor, six first‐order factors	634.75	146	0.85	0.83	0.08	50,978.79	51,244.05
A4	Bifactor model: One general, five specific^a^	626.34	128	0.85	0.80	0.09	50,959.78	51,300.84
Data‐driven exploratory factor analysis								
B1	One factor model	1231.15	152	0.67	0.63	0.12	51,687.27	51,927.27
B2	Two factor model	880.07	134	0.77	0.71	0.11	51,260.09	51,575.88
B3	Three factor model	638.04	117	0.84	0.77	0.10	50,966.21	51,353.57
B4	Four factor model	353.14	101	0.92	0.87	0.07	50,711.00	51,165.75
B5	Five factor model	244.75	86	0.95	0.90	0.06	50,620.05	51,137.95
B6	Bifactor model: One general, one specific	880.07	134	0.77	0.71	0.11	51,260.09	51,575.88
B7	Bifactor model: One general, two specific	638.04	117	0.84	0.77	0.10	50,966.21	51,353.57
B8	Bifactor model: One general, three specific	353.14	101	0.92	0.87	0.07	50,711.00	51,165.75
B9	Bifactor model: One general, four specific	244.75	86	0.95	0.90	0.06	50,620.05	51,137.95
CFA of models from exploratory factor analysis								
C1	Five factor solution (initial)	400.94	130	0.92	0.89	0.07	50,730.32	51,062.95
C2	Bifactor model solution (initial)	455.22	134	0.90	0.88	0.07	50,788.49	51,104.29
C3	Bifactor model solution with correlated residuals	334.39	129	0.94	0.92	0.06	50,656.84	50,993.69
C4	Bifactor model solution with correlated residuals (S2)	381.47	129	0.93	0.90	0.06	50,573.49	50,910.17
C5	Bifactor model solution with correlated residuals (all)	569.19	129	0.93	0.91	0.06	101,215.99	101,608.21

*Note*: C3. Modification indices suggested that the error terms for the following five pairs of items were correlated: Reading Difficulties and Word Finding Difficulties (ARQ), Sensory Sensitivity (GSQ) and Heightened Visual Sensitivity (CHI‐II), Fine Motor Difficulties (ADC‐R) and Reading Difficulties (ARQ), Sensory Sensitivity (GSQ) and Preference for Numbers and Patterns (AQ), Distorted Visual Perception (CHI‐II) and Hyperactivity (CAARS).

^a^ A bifactor model with one general factor and six specific factors did not converge.

**FIGURE 2:**
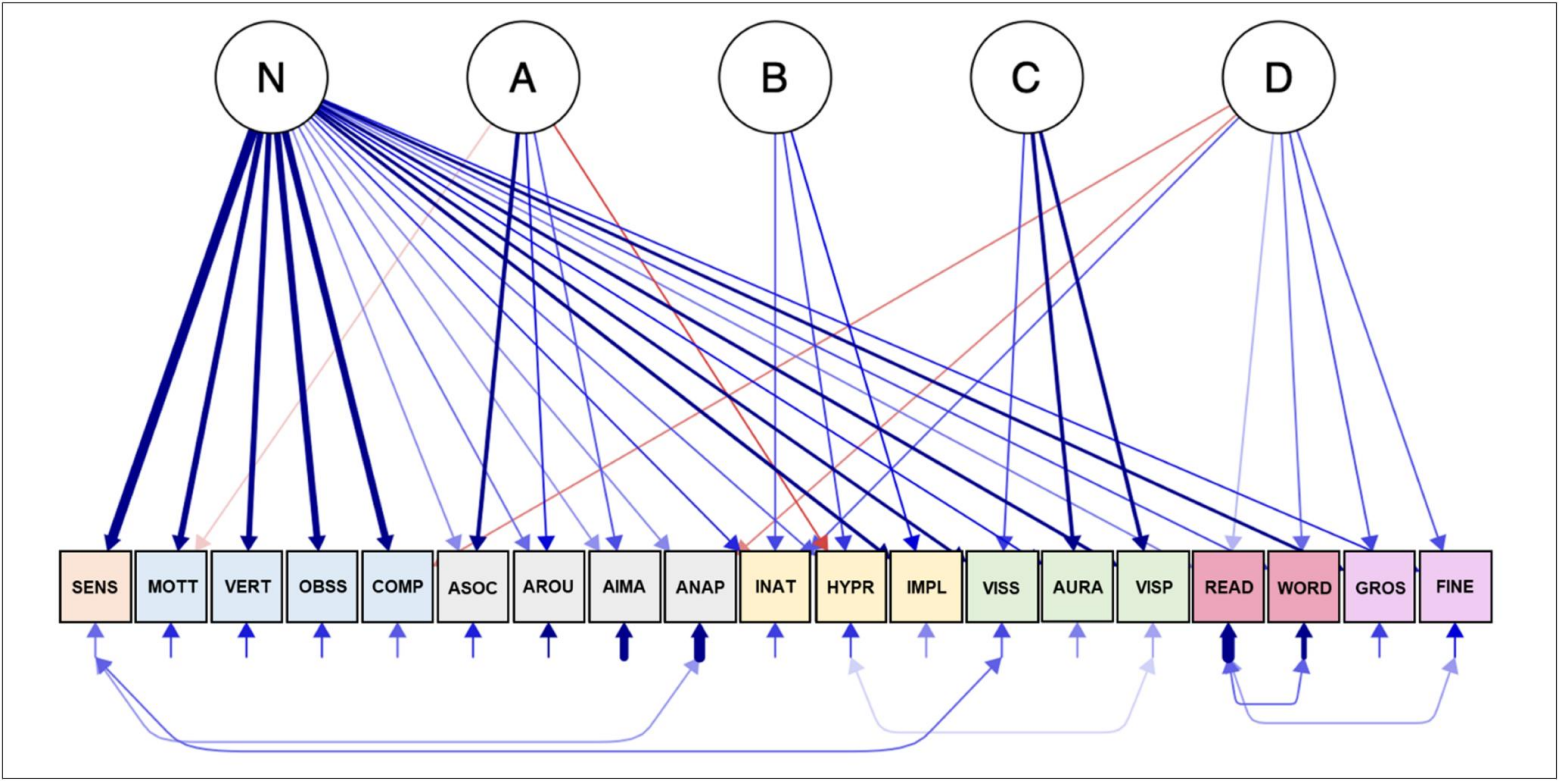
Path Diagram Depicting Final Bifactor Model. Blue lines indicate positive loadings and red lines indicate negative loadings. Thicker lines depict stronger loadings. Standardized loadings and residual covariances are shown in Table S2 (Supporting Information). SENS = GSQ Sensory Sensitivities. MOTT = MOVES Motor Tics. VERT = MOVES Verbal Tics. OBSS = MOVES Obsessions. COMP = MOVES Compulsions. ASOC = AQ Social Skills Difficulties. AROU = AQ Preference for Routines. AIMA = AQ Imagination. ANAP = AQ Numbers and Patterns. INAT = CAARS Inattentiveness. HYPR = CAARS Hyperactivity. IMPL = CAARS Impulsivity. VISS = CHI‐II Heightened Visual Sensitivity. AURA = CHI‐II Aura‐Like Hallucinatory Experiences. VISP = CHI‐II Distorted Visual Perception. READ = ARQ Reading Difficulties. WORD = ARQ Word Finding Difficulties. GROS = ADC‐R Gross Motor Skills. FINE = ADC‐R Fine Motor Skills.

CFA of the 5‐factor model specification (with no cross‐loadings) provided a poor fit to the data (Table 2). Modification indices suggested that there were correlated residual terms. We respecified the model to estimate these parameters but this model resulted in unacceptable parameter estimates as indicated by a negative residual variance estimate. CFA using the bifactor model specification provided an initially poor fit to the model. Re‐specification of the model based on the modification indices improved the overall model fit (Table 2). We cross‐validated the re‐specified bifactor model in the hold‐out sample and it fit the data adequately. A simplified path diagram of this final model is shown in Figure 2. The standardized loadings can be found in the Supporting Information (Table S3).

The bifactor model results indicated that individual differences in neurodiversity characteristics/traits were explained by both a general neurodiversity factor (that we called “N”) and four orthogonal specific factors (that we called A, B, C, D). The N factor captured covariance in traits that crosscut the original condition‐specific broader phenotype scales. Factor loadings indicated that several characteristics (e.g., sensory sensitivities, verbal tics, obsessive behaviour) were not unique to any specific broader phenotype, loading only on the N factor. A model‐based estimate of reliability, *ω*_H_ = 0.81, indicated that 81% of the variance in summed total scores across the original scales was accounted for by the N Factor (Rodriguez et al., 2016).

The four specific factors captured common variance distinct from the general N factor in subsets of traits typically associated with specific neurodevelopmental conditions. Factor A explained variance in characteristics typically associated with autism. High scores on this latent factor were linked with lower social and imaginative abilities, a preference for routines, and low levels of hyperactivity and motor tics. Factor B explained variance in characteristics associated with ADHD. High scores were linked with inattentiveness, hyperactivity, and impulsivity. Factor C explained variance in cortical hyperexcitability. High scores on this factor were linked with visual sensitivity, aura‐like experiences, and visual perceptual distortions. Factor D captured characteristics associated with dyslexia and dyspraxia. High scores were linked with difficulties with reading, fine and gross‐motor coordination, and inattentiveness, as well as low levels of compulsive behaviour and reduced preference for numbers and patterns. Model‐based estimates of reliability indicated that, once variance due to N was taken into account, the percentage of variance in summed subscale scores accounted for by the specific factors was 39% for Factor A, *ω*_HS_ = 0.39, 38% for Factor B, *ω*_HS_ = 0.38, 42% for Factor C, *ω*_HS_ = 0.42, and 25% for Factor D, *ω*_HS_ = 0.25 (Rodriguez et al., 2016). Note that, for some items, there were correlated residual terms that cross‐cut specific latent factors (e.g., hyperactivity and distorted visual perception). These correlated residual terms indicate that there was variance in these items that was not explained by either the general or specific latent factors but was shared between these items. The general and specific latent factors were entirely orthogonal (i.e., the correlation between these factors was fixed at 0).

### Participant characteristics and neurodiversity

We first examined the association between the general and specific neurodiversity latent factors and participants' socio‐demographic characteristics by regressing each factor onto indicators for age, gender (0 = man, 1 = woman) and self‐reported socio‐economic status (see Supporting Information Table S2 for model fit). Compared with younger participants, older participants scored lower on the N Factor, *β* = −0.28, SE = 0.03, *p* < 0.0001, and B Factor, *β* = −0.09, SE = 0.04, *p* < 0.05, but higher on the A Factor, *β* = 0.27, SE = 0.04, *p* < 0.0001. Higher socio‐economic status was negatively associated with scores on the N Factor, *β* = −0.15, SE = 0.03, *p* < 0.0001, the A Factor, *β* = −0.16, SE = 0.04, *p* < 0.0001, and the D Factor, *β* = −0.11, SE = 0.04, *p* < 0.05. There were no gender differences on the N Factor but, on average, women had higher scores than men on the D Factor, *β* = 0.36, SE = 0.04, *p* < 0.0001, and men had higher scores than women on the B Factor, *β* = −0.40, SE = 0.04, *p* < 0.0001.

### Executive function, cognitive ability and neurodiversity

Next, we examined the links between the general and specific neurodiversity latent factors, executive function (i.e., strategic planning, organisation, impulse control, empathy) and non‐verbal intelligence. We regressed each indicator of executive function and non‐verbal intelligence onto the general and specific neurodiversity factors, age, gender and socio‐economic status (Supporting Information Table S2). The dependent variables (i.e., executive function and non‐verbal intelligence indicators) were permitted to correlate with each other. The predictor variables (except for the orthogonal factors) were also permitted to covary in the model. The results of the model are shown in Table 3. Although the N Factor was associated with lower empathy and organisation, stronger links were observed between high scores on the A Factor and lower empathy, and high scores on the B factor and lower impulse control. The N factor was not associated with strategic planning, impulse control, or non‐verbal intelligence. Instead, high scores on the A Factor were associated with better planning while high scores on the B factor were associated with poorer planning and somewhat poorer performance on the non‐verbal intelligence task. High scores on the D Factor were associated with better empathy but weaker organisational skills. Factor C was weakly associated with impulse control difficulties and somewhat poorer performance on the non‐verbal intelligence task.

**TABLE 3:** Standardized robust maximum likelihood (MLR) estimates for relations between neurodiversity factors, executive function and non‐verbal ability.

	Empathy		Strategic planning		Organisation		Impulse control		Non‐verbal ability	
	***β***	SE	***β***	SE	***β***	SE	***β***	SE	***β***	SE
N Factor	−0.15***	0.04	−0.03	0.04	−0.33***	0.03	−0.08	0.07	−0.05	0.04
A factor	−0.37***	0.04	0.11*	0.04	0.04	0.03	0.18***	0.03	0.05	0.04
B factor	−0.17***	0.04	−0.20***	0.04	−0.02	0.03	−0.53***	0.04	−0.11**	0.04
C factor	−0.05	0.04	0.02	0.04	−0.01	0.03	−0.09**	0.03	−0.11**	0.04
D factor	0.11**	0.04	0.01	0.05	−0.19***	0.04	0.03	0.04	0.04	0.05
Age	0.12***	0.03	0.02	0.03	0.11***	0.02	0.21***	0.02	−0.05	0.04
Gender	0.04	0.05	0.04	0.04	0.02	0.03	−0.14**	0.05	−0.13**	0.04
SES	−0.02	0.03	0.23***	0.03	−0.02	0.02	0.03	0.02	0.05	0.03

*Note*: A = Characteristics related to autism. B = Characteristics related to ADHD. C = Characteristics linked with cortical hyperexcitability. D = Characteristics linked with dyslexia and dyspraxia.

***p < 0.001. **p < 0.01. *p < 0.05.

### Mental health, wellbeing and neurodiversity

Finally, we examined the links between the neurodiversity latent factors and participants' mental health and wellbeing. We regressed indicators of depressive symptoms, anxiety symptoms, and mental wellbeing onto the general and specific neurodiversity factors, age, gender, socio‐economic status and non‐verbal ability (Supporting Information Table S2). The dependent variables (i.e., mental health and wellbeing indicators) were permitted to covary. The predictor variables (except for the orthogonal factors) were also permitted to covary in The model. The results of the model are depicted in Table 4. High scores on the N Factor were associated with lower wellbeing and higher levels of anxiety and depressive symptoms. High scores on the A and B Factors were also associated with lower wellbeing and weakly associated with higher anxiety and depressive symptoms. Scores on the C and D Factors were unrelated to anxiety or depressive symptoms.

**TABLE 4:** Standardized robust maximum likelihood (MLR) estimates for relations between neurodiversity factors, wellbeing and mental health.

	Wellbeing		Depressive symptoms		Anxiety symptoms	
	***β***	SE	***β***	SE	***β***	SE
N Factor	−0.36***	0.03	0.52***	0.03	0.55***	0.03
A factor	−0.42***	0.04	0.18***	0.04	0.13***	0.04
B factor	−0.18***	0.04	0.19***	0.04	0.28***	0.04
C factor	0.02	0.04	0.04	0.04	0.05	0.04
D factor	−0.12**	0.04	0.08	0.04	−0.05	0.04
Age	0.18***	0.03	−0.13***	0.03	−0.12***	0.03
Gender	−0.08*	0.04	0.14***	0.04	0.24***	0.04
SES	0.20***	0.03	−0.14***	0.03	−0.12***	0.03
Non‐verbal ability	−0.03	0.03	0.02	0.02	0.004	0.02

*Note*: A = Characteristics related to autism. B = Characteristics related to ADHD. C = Characteristics linked with cortical hyperexcitability. D = Characteristics linked with dyslexia and dyspraxia.

***p < 0.001. **p < 0.01. *p < 0.05.

## DISCUSSION

We conducted a pre‐registered series of theory‐driven and data‐driven analyses of our data on the broader phenotypes associated with six neurodevelopmental conditions (autism, ADHD, dyslexia, dyspraxia, tic disorders/Tourette's, cortical hyperexcitability associated with epilepsy) plus sensory sensitivities. Theory‐driven analyses tested alternative models of co‐variance between broader phenotype measures, either while preserving the grouping of subscales according to traditional neurodevelopmental conditions, or else dispensing entirely with these groupings. No model provided a good fit for the data. Data‐driven analyses allowed subscales to re‐group into new factors, with the N + 4 model providing the best fit. As per our pre‐registered plan, we cross‐validated this model in an entirely new set of participants, providing confirmatory evidence that the N + 4 model captures the structure of neurodiversity in representative samples from the UK population. Below we discuss the implications of these results.

### Factor structure: Patterns of similarity and diversity

Given that each of the original scales was devised to assess the broader phenotype of a particular neurodevelopmental condition, the status quo position for examining similarity and diversity between these phenotypes should retain the structure of the original scales grouped by condition. The fact that a simpler N + 4 factor model provided a better fit for the data suggests that there is overlap in some of the traits assessed in the original measures, which can be summarised more effectively with fewer factors. The best model included a general N factor that accounted for individual differences in traits that cut across all neurodevelopmental phenotypes assessed. Individual differences in some traits were also explained by specific factors A‐D reflecting characteristics associated with autism (Factor A), ADHD (Factor B), cortical hyperexcitability (Factor C), and dyslexia/dyspraxia (Factor D). These features of the N + 4 model capture the sense that there is considerable overlap in the broader phenotypes associated with different neurodevelopmental conditions and that the same traits can be explained by different underlying causes when they load on both the general factor and a specific factor.

However, a further critical feature of the N + 4 model is that its five factors are orthogonal, meaning that they can vary independently of one another. It is informative to contrast this with the “status quo” approach of studying broader phenotypes of neurodiversity by simply using a combination of existing scales for different phenotypes. The descriptive power of such an approach is severely limited by the high correlations between scales, which mean (for example) that a person with a high score on any one scale is likely to have a high score on every other scale. This makes it very difficult for a simple combination of these scales to do justice to the apparent complexity of phenotypes described in the introduction. In contrast the orthogonality of factors in the N + 4 model gives much greater descriptive power by characterising each person on five statistically independent latent dimensions.

To illustrate, this means that two people who score high on Factor A could equally well show different profiles across the other four factors. One person might combine lower social skills and imagination and a preference for routines (Factor A) with high inattentiveness, hyperactivity, and impulsivity (Factor B), while the other person might combine the same Factor A traits with high difficulties with reading, fine and gross‐motor coordination (and/or traits loading on Factor C) but low traits for Factor B. Of course, the factor structure does also constrain the likely phenotypes: a person who scores high on Factor A is likely to report more difficulties with social skills, social imagination, and a greater preference for routines, because all three of these subscales load highly onto Factor A.

In summary, the orthogonal N + 4 factor structure maximises the differentiation of traits that covary and traits that do not. In doing so it helps cast light on the “complexity” of neurodiversity broader phenotypes by identifying sources that account for both the similarities between notionally distinct phenotypes (the N factor), and sources that can account for highly distinctive patterns of difference between phenotypes.

### Redundancy, relevance, and selectivity

#### Redundancy

We found no evidence that any of the N + 4 factors could be explained in terms of general cognitive abilities (non‐verbal ability, or executive function). Although general cognitive abilities can explain a large amount of variance in many aspects of human behaviour (e.g., Friedman & Miyake, 2017; Mackintosh, 2011), they did not share the majority of the variance measured by the N + 4 factors. *Relevance.* We found evidence that the N + 4 factors measured variance that was related to other characteristics, for wellbeing, depression, and anxiety, with the N factor showing particularly strong relationships to these measures. Importantly, these relationships also showed *selectivity*. All N + 4 factors predicted at least one aspect of executive function and/or wellbeing, depression, or anxiety, but none predicted all of them. For example, Factors C and D did not predict depression or anxiety symptoms, and even though the N factor strongly predicted depression and anxiety symptoms, it only predicted 2 of the 4 aspects of executive function. This observation suggests that the factors are not only statistically independent but that they are differentially sensitive to variation in characteristics and experiences that are important in everyday life.

### Interpretation of the factors, and implications for understanding neurodevelopmental conditions

We must emphasise that the present study examined neurodiversity broader phenotypes in a representative sample from the whole adult population of the UK. While 3.23% of our sample said they had a formally diagnosed neurodevelopmental condition, and a further 12.97% were self‐diagnosed, further work would be necessary to test whether the conclusions from the present study apply specifically to either of these groups. With that major caveat we will proceed cautiously to highlight potential implications based on research into the factor structure of psychiatric and neurodevelopmental conditions.

We find evidence of a general N‐factor that accounts for a large amount of variance across all neurodiversity characteristics. From prior work on “p factor”, we might expect the N factor to predict the “severity” of difficulties experienced (regardless of their nature), and the likelihood of multiple diagnoses now, or in the future. The factor structure might help explain why high scores on broader phenotype measures are not consistently related to diagnostic status (e.g., Abu‐Akel et al., 2019; Lundqvist & Lindner, 2017). For example, it is conceivable that a high level on one specific factor (A,B,C, or D) is necessary but not sufficient for diagnosis. The likelihood of diagnosis may depend in addition on a co‐occurring high level on the N factor level.

The orthogonal factor structure also allows for individuals' trait profiles to arise for multiple reasons. For example, it seems likely that autistic people would tend to have high scores on the A factor. The fact that some (but not all) autistic people experience sensory sensitivity or impulsivity might be accounted for by the N factor, because those traits load onto the N factor not the A factor. Likewise, the observation that people with dyslexia sometimes experience difficulties associated with ADHD could be explained by the combination of high C‐factor and either high B factor or high N factor, since traits relevant to ADHD load strongly onto both of the latter factors. Finally, the same possibilities would make it very plausible that people might meet diagnostic criteria for a given condition with different combinations of the underlying factors. Examining these possibilities directly clearly requires further work. We hope that here we have illustrated the potential for the N + 4 model to help do justice to some of the phenotypic complexity that is widely seen to be a recurrent feature of neurodevelopmental conditions (Astle et al., 2022; Dwyer, 2022; Embracing complexity, 2021; Fletcher‐Watson, 2022).

### Factor structure as a “signature”

While it is possible that the factor structure of a majority‐neurotypical sample may not apply in a sample of neurodivergent people, the approach taken here provides promising tools for operationalising this and other questions about variation in neurodiversity. Researchers, clinicians, and people with lived experience of neurodivergence are increasingly questioning how neurodevelopmental conditions are experienced and diagnosed in people of different genders, ages, ethnicities, or cultural backgrounds (Happé & Frith, 2020; Macdonald & Deacon, 2019; Mandell et al., 2009). The current approach can help address these questions by examining the “measurement invariance” of the factor structure over different groups. This has the potential to reveal whether the same underlying structure exists even for different groups who might experience different levels of difficulty (perhaps neurotypical vs. neurodivergent groups), and also highlight potential limitations of measures that may be more suitable or sensitive to the experiences of one group (such as males vs. females).

### What do “broader phenotype measures” measure?

The present work has implications for our understanding of broader phenotypes of neurodevelopmental conditions, and the practice of examining them with scales designed to capture the phenotypes of specific conditions. Put simply, our findings suggest that each individual broader phenotype scale mostly measures “neurodiversity”: variability that is general, rather specifically associated with any one neurodevelopmental condition. Some scales also measure variability in traits that are linked to particular neurodevelopmental conditions, but this is only apparent when the scales are analysed together, allowing the general and specific variance to be distinguished. We suggest that future work would benefit from adopting the use of multiple scales linked to different neurodevelopmental conditions, and examining individual differences in latent factors, as in the N + 4 model, rather than scale‐specific scores.

### Assumptions, limitations, and further work

Our approach reflects a common practice of distinguishing between neurodevelopmental and psychiatric conditions. One consequence was that our selection of broader phenotype measures prioritised conditions that most people would agree were “neurodevelopmental”. A second consequence was that we explored links between neurodiversity traits and self‐rated depression, anxiety, and mental wellbeing, rather than testing how they might relate within a common factor structure. This approach is in accord with claims that the experience of neurodivergence in a majority‐neurotypical world can have negative consequences for mental health and wellbeing (Alexander‐Passe, 2015; Cage et al., 2018; Cage & Troxell‐Whitman, 2019; Dwyer, 2022; Gallant & Good, 2023; Kiraz & Sertçelik, 2021; Mantzalas et al., 2022; Pryke‐Hobbes et al., 2023; Reindal, 2008). However, as noted in the introduction, the distinction between neurodevelopmental and psychiatric conditions may be questioned in at least some cases. Future work incorporating measures of psychosis, internalizing and internalizing will shed light on the distinctiveness of the N and p factors. Finally, any study of this kind must make pragmatic decisions about the number of measures to include, and therefore the number of broader phenotypes that are represented in the data. Our selection of 7 broader phenotype measures corresponded to some of the most frequent neurodevelopmental conditions (e.g. as identified by Cleaton & Kirby, 2018, Zablotsky et al., 2019, Francés et al., 2022, Straub et al., 2022), and 6/7 had two or more subscales. The present study is not an exhaustive representation of “neurodiversity”, though given the contested boundaries between “neurodevelopmental” and “psychiatric” conditions it is not currently clear what ought to be included in an attempt at such an exhaustive project.

Future work is necessary to examine the relationships between broader phenotypes for a larger set of neurodevelopmental and psychiatric conditions. It is equally important to test the invariance of factor structures over different measures of the same putative phenotype. It cannot be taken for granted that the same general and specific factor structure will emerge when different measures are employed. The most powerful evidence would come from convergence between findings from self‐assessments of the kind employed here and those from third‐party informants (such as friends, parents, teachers, or clinicians). Ratings from third‐party informants would make it possible to examine the broader phenotypes of children who may not be able to give reliable self‐ratings and would make it possible to test whether the factor structure changed over development. Finally, it is important to recognise that longitudinal research is uniquely informative about the causal direction of statistical associations. For example, it is conceivable that the best statistical models of cross‐sectional data do not support a clear distinction between neurodevelopmental and psychiatric conditions or broader phenotypes. Nonetheless it could be that longitudinal data still reveal causal priority, for example, showing that earlier neurodiversity traits predict later mental wellbeing more strongly than either the reverse pattern, or concurrent relationships at any one point in time. Such findings would have implications for individual and environmental interventions to support mental wellbeing, and whether they could be tailored to take account of the neurodiversity of individuals or groups.

## CONCLUSION

In a representative sample of 995 adults, the N + 4 model best accounted for individual differences in broader phenotype traits related to 6 neurodevelopmental conditions. A large amount of variance was accounted for by a general N Factor that cut across all the original condition‐specific measures, and further variance was accounted for by 4 specific factors were more strongly linked to features commonly associated with specific neurodevelopmental conditions. We believe this is a promising approach to studying the “complexity” of neurodiversity phenotypes. The current results have direct implications for future studies of broader phenotypes across the whole population of neurotypical and neurodivergent people, and suggest novel, informative, and tractable ways of addressing related questions specifically in relation to neurodevelopmental conditions.

## AUTHOR CONTRIBUTIONS

**Ian A. Apperly**: Conceptualization; funding acquisition; supervision; writing – original draft; writing – review & editing. **Robert Lee**: Data curation; investigation; methodology; project administration; writing – original draft; writing – review & editing. **Sanne W. van der Kleij**: Methodology; supervision; writing – review & editing. **Rory T. Devine**: Conceptualization; formal analysis; funding acquisition; supervision; visualization; writing – original draft; writing – review & editing.

## CONFLICT OF INTEREST STATEMENT

The authors have declared they have no competing or potential conflicts of interest.

### OPEN RESEARCH BADGES

This article has earned a Preregistered Research Designs badge for having a preregistered research design, available at https://osf.io/rtsjy.

## Ethical considerations

Approval was obtained from the University of Birmingham Science, Technology, Engineering, and Maths Ethics Committee (ERN 22‐1192).

## Supporting information

Supplementary Information S1

## Data Availability

Funding for this research mandates open publication of data with the UK Data Service.

## ENDNOTES

^1^
The concept of neurodiversity has other important motivations and implications (for recent review and discussion see Dwyer, 2022). Here we focus on the two that are particularly relevant for the present research.

^2^
For brevity we will use “phenotype” to refer both to characteristics that are observable “from the outside” and the lived experience of individual people.

## References

[jcv212219-bib-0001] Abu‐Akel, A. , Allison, C. , Baron‐Cohen, S. , & Heinke, D. (2019). The distribution of autistic traits across the autism spectrum: Evidence for discontinuous dimensional subpopulations underlying the autism continuum. Molecular Autism, 10, 1–13. 10.1186/s13229-019-0275-3 31149329 PMC6537408

[jcv212219-bib-0002] Addicoat, A. , Thapar, A. K. , Riglin, L. , Thapar, A. , & Collishaw, S. (2020). Adult mood problems in children with neurodevelopmental problems: Evidence from a prospective birth cohort followed to age 50. Social Psychiatry and Psychiatric Epidemiology, 55(3), 351–358. 10.1007/s00127-019-01727-5 31119307

[jcv212219-bib-0003] Adler, N. E. , Epel, E. S. , Castellazzo, G. , & Ickovics, J. R. (2000). Relationship of subjective and objective social status with psychological and physiological functioning: Preliminary data in healthy, White women. Health Psychology, 19(6), 586–592. 10.1037/0278-6133.19.6.586 11129362

[jcv212219-bib-0004] Alexander‐Passe, N. (2015). Dyslexia and Mental Health: Helping people identify destructive behaviours and find positive ways to cope. Jessica Kingsley Publishers.

[jcv212219-bib-0005] American Psychiatric Association . (2022). Neurodevelopmental disorders. In Diagnostic and statistical manual of mental disorders (5th ed., pp. 31–86). *text rev*.

[jcv212219-bib-0006] Astle, D. E. , Holmes, J. , Kievit, R. , & Gathercole, S. E. (2022). Annual Research Review: The transdiagnostic revolution in neurodevelopmental disorders. Journal of Child Psychology and Psychiatry, 63(4), 397–417. 10.1111/jcpp.13481 34296774

[jcv212219-bib-0007] Baron‐Cohen, S. , Wheelwright, S. , Skinner, R. , Martin, J. , & Clubley, E. (2001). The autism‐spectrum quotient (AQ): Evidence from asperger syndrome/high‐functioning autism, males and females, scientists and mathematicians. Journal of Autism and Developmental Disorders, 31(1), 5–17. 10.1023/a:1005653411471 11439754

[jcv212219-bib-0008] Berl, M. M. , Terwilliger, V. , Scheller, A. , Sepeta, L. , Walkowiak, J. , & Gaillard, W. D. (2015). Speed and complexity characterize attention problems in children with localization‐related epilepsy. Epilepsia, 56(6), 833–840. 10.1111/epi.12985 25940056 PMC4457628

[jcv212219-bib-0009] Bilker, W. B. , Hansen, J. A. , Brensinger, C. M. , Richard, J. , Gur, R. E. , & Gur, R. C. (2012). Development of abbreviated nine‐item forms of the Raven’s standard progressive matrices test. Assessment, 19(3), 354–369. 10.1177/1073191112446655 22605785 PMC4410094

[jcv212219-bib-0010] Birnbaum, R. , & Weinberger, D. R. (2017). Genetic insights into the neurodevelopmental origins of schizophrenia. Nature Reviews Neuroscience, 18(12), 727–740. 10.1038/nrn.2017.125 29070826

[jcv212219-bib-0011] Bloemen, A. J. P. , Oldehinkel, A. J. , Laceulle, O. M. , Ormel, J. , Rommelse, N. N. J. , & Hartman, C. A. (2018). The association between executive functioning and psychopathology: General or specific? Psychological Medicine, 48(11), 1787–1794. 10.1017/s0033291717003269 29521611

[jcv212219-bib-0012] Blume, H. (1998). Neurodiversity: On the neurological underpinnings of geekdom. Atlantic. Available at www.theatlantic.com/magazine/archive/1998/09/neurodiversity/305909/

[jcv212219-bib-0013] Brown, T. A. (2015). Confirmatory factor analysis for applied research. Guilford publications.

[jcv212219-bib-0014] Cage, E. , Di Monaco, J. , & Newell, V. (2018). Experiences of autism acceptance and mental health in autistic adults. Journal of Autism and Developmental Disorders, 48(2), 473–484. 10.1007/s10803-017-3342-7 29071566 PMC5807490

[jcv212219-bib-0015] Cage, E. , & Troxell‐Whitman, Z. (2019). Understanding the reasons, contexts and costs of camouflaging for autistic adults. Journal of Autism and Developmental Disorders, 49(5), 1899–1911. 10.1007/s10803-018-03878-x 30627892 PMC6483965

[jcv212219-bib-0016] Caspi, A. , Houts, R. M. , Belsky, D. W. , Goldman‐Mellor, S. J. , Harrington, H. , Israel, S. , Meier, M. H. , Ramrakha, S. , Shalev, I. , Poulton, R. , & Moffitt, T. E. (2014). The p factor: One general psychopathology factor in the structure of psychiatric disorders? Clinical Psychological Science, 2(2), 119–137. 10.1177/2167702613497473 25360393 PMC4209412

[jcv212219-bib-0017] Caspi, A. , & Moffitt, T. (2018). All for one and one for all: Mental disorders in one dimension. American Journal of Psychiatry, 175(9), 831–844. 10.1176/appi.ajp.2018.17121383 29621902 PMC6120790

[jcv212219-bib-0018] Chaix, Y. , Albaret, J. M. , Brassard, C. , Cheuret, E. , De Castelnau, P. , Benesteau, J. , Karsenty, C. , Démonet, J. F. , & Démonet, J. F. (2007). Motor impairment in dyslexia: The influence of attention disorders. European Journal of Paediatric Neurology, 11(6), 368–374. 10.1016/j.ejpn.2007.03.006 17467315

[jcv212219-bib-0019] Cleaton, M. A. M. , & Kirby, A. (2018). Why do we find it so hard to calculate the burden of neurodevelopmental disorders? Journal of Childhood & Developmental Disorders, 4(3), 1–20. 10.4172/2472-1786.100073

[jcv212219-bib-0020] Conners, C. K. , Erhardt, D. , & Sparrow, M. A. (2003). Conners’ adult ADHD rating scales (CAARS). Archives of Clinical Neuropsychology, 18(4), 431–437. 10.1016/s0887-6177(03)00021-0

[jcv212219-bib-0021] Danielson, M. L. , Bitsko, R. H. , Ghandour, R. M. , Holbrook, J. R. , Kogan, M. D. , & Blumberg, S. J. (2018). Prevalence of parent‐reported ADHD diagnosis and associated treatment among US children and adolescents, 2016. Journal of Clinical Child and Adolescent Psychology, 47(2), 199–212. 10.1080/15374416.2017.1417860 29363986 PMC5834391

[jcv212219-bib-0022] DuPaul, G. J. , Gormley, M. J. , & Laracy, S. D. (2013). Comorbidity of LD and ADHD: Implications of DSM‐5 for assessment and treatment. Journal of Learning Disabilities, 46(1), 43–51. 10.1177/0022219412464351 23144063

[jcv212219-bib-0023] Dwyer, P. (2022). The neurodiversity approach (es): What are they and what do they mean for researchers? Human Development, 66(2), 73–92. 10.1159/000523723 36158596 PMC9261839

[jcv212219-bib-0024] Eberhard, D. , Billstedt, E. , & Gillberg, C. (2022). Neurodevelopmental disorders and comorbidity in young adults attending a psychiatric outpatient clinic. Psychiatry Research, 313, 114638. 10.1016/j.psychres.2022.114638 35597136

[jcv212219-bib-0025] Embracing Complexity . (2021). Embracing complexity in research. Retrieved from https://embracingcomplexity.org.uk/reports

[jcv212219-bib-0026] England‐Mason, G. (2020). Emotion regulation as a transdiagnostic feature in children with neurodevelopmental disorders. Current Developmental Disorders Reports, 7(3), 130–138. 10.1007/s40474-020-00200-2

[jcv212219-bib-0027] Farran, E. K. , Bowler, A. , D’Souza, H. , Mayall, L. , Karmiloff‐Smith, A. , Sumner, E. , Brady, D. , & Hill, E. L. (2020). Is the motor impairment in attention deficit hyperactivity disorder (ADHD) a co‐occurring deficit or a phenotypic characteristic? Advances in Neurodevelopmental Disorders, 4(3), 253–270. 10.1007/s41252-020-00159-6

[jcv212219-bib-0028] Fletcher‐Watson, S. (2022). Transdiagnostic research and the neurodiversity paradigm. Journal of Child Psychology and Psychiatry, 63(4), 418–420. 10.1111/jcpp.13589 35187674 PMC9303713

[jcv212219-bib-0029] Fong, C. Y. , Takahashi, C. , & Braithwaite, J. J. (2019). Evidence for distinct clusters of diverse anomalous experiences and their selective association with signs of elevated cortical hyperexcitability. Consciousness and Cognition, 71, 1–17. 10.1016/j.concog.2019.03.003 30904823

[jcv212219-bib-0030] Francés, L. , Quintero, J. , Fernández, A. , Ruiz, A. , Caules, J. , Fillon, G. , Hervás, A. , & Soler, C. V. (2022). Current state of knowledge on the prevalence of neurodevelopmental disorders in childhood according to the DSM‐5: A systematic review in accordance with the PRISMA criteria. Child and Adolescent Psychiatry and Mental Health, 16(1), 27. 10.1186/s13034-022-00462-1 35361232 PMC8973738

[jcv212219-bib-0031] Friedman, N. P. , & Miyake, A. (2017). Unity and diversity of executive functions: Individual differences as a window on cognitive structure. Cortex, 86, 186–204. 10.1016/j.cortex.2016.04.023 27251123 PMC5104682

[jcv212219-bib-0032] Gaffney, G. R. , Sieg, K. , & Hellings, J. (1994). The MOVES: A self‐rating scale for tourette's syndrome. Journal of Child and Adolescent Psychopharmacology, 4(4), 269–280. 10.1089/cap.1994.4.269

[jcv212219-bib-0033] Gallant, C. , & Good, D. (2023). Predictors of mental health Service use among children and adolescents with and without neurodevelopmental disorders. Journal of Mental Health Research in Intellectual Disabilities, 16(2), 142–161. 10.1080/19315864.2022.2105996

[jcv212219-bib-0034] Ghacibeh, G. A. , & Fields, C. (2015). Interictal epileptiform activity and autism. Epilepsy and Behavior, 47, 158–162. 10.1016/j.yebeh.2015.02.025 25847431

[jcv212219-bib-0035] Happé, F. , & Frith, U. (2020). Annual Research Review: Looking back to look forward–changes in the concept of autism and implications for future research. Journal of Child Psychology and Psychiatry, 61(3), 218–232. 10.1111/jcpp.13176 31994188

[jcv212219-bib-0036] Hirschtritt, M. E. , Lee, P. C. , Pauls, D. L. , Dion, Y. , Grados, M. A. , Illmann, C. , King, R. A. , Sandor, P. , McMahon, W. M. , Lyon, G. J. , Cath, D. C. , Kurlan, R. , Robertson, M. M. , Osiecki, L. , Scharf, J. M. , & Mathews, C. A. , & Tourette Syndrome Association International Consortium for Genetics . (2015). Lifetime prevalence, age of risk, and genetic relationships of comorbid psychiatric disorders in Tourette syndrome. JAMA Psychiatry, 72(4), 325–333. 10.1001/jamapsychiatry.2014.2650 25671412 PMC4446055

[jcv212219-bib-0037] Hoekstra, R. A. , Vinkhuyzen, A. A. , Wheelwright, S. , Bartels, M. , Boomsma, D. I. , Baron‐Cohen, S. , Posthuma, D. , & Van Der Sluis, S. (2011). The construction and validation of an abridged version of the autism‐spectrum quotient (AQ‐Short). Journal of Autism and Developmental Disorders, 41(5), 589–596. 10.1007/s10803-010-1073-0 20697795 PMC3076581

[jcv212219-bib-0038] Hofvander, B. , Delorme, R. , Chaste, P. , Nydén, A. , Wentz, E. , Ståhlberg, O. , Herbrecht, E. , Stopin, A. , Anckarsäter, H. , Gillberg, C. , Råstam, M. , & Leboyer, M. (2009). Psychiatric and psychosocial problems in adults with normal‐intelligence autism spectrum disorders. BMC Psychiatry, 9(1), 1–9. 10.1186/1471-244x-9-35 19515234 PMC2705351

[jcv212219-bib-0039] Howlin, P. , & Magiati, I. (2017). Autism spectrum disorder: Outcomes in adulthood. Current Opinion in Psychiatry, 30(2), 69–76. 10.1097/yco.0000000000000308 28067726

[jcv212219-bib-0040] Katzman, M. A. , Bilkey, T. S. , Chokka, P. R. , Fallu, A. , & Klassen, L. J. (2017). Adult ADHD and comorbid disorders: Clinical implications of a dimensional approach. BMC Psychiatry, 17, 1–15. 10.1186/s12888-017-1463-3 28830387 PMC5567978

[jcv212219-bib-0041] Kiraz, S. , & Sertçelik, S. (2021). Adult attention deficit hyperactivity disorder and early maladaptive schemas. Clinical Psychology & Psychotherapy, 28(5), 1055–1064. 10.1002/cpp.2569 33586830

[jcv212219-bib-0042] Kirby, A. , Edwards, L. , Sugden, D. , & Rosenblum, S. (2010). The development and standardization of the adult developmental co‐ordination disorders/dyspraxia checklist (ADC. Research in Developmental Disabilities, 31(1), 131–139. 10.1016/j.ridd.2009.08.010 19819107

[jcv212219-bib-0043] Kroenke, K. , Spitzer, R. L. , & Williams, J. B. (2001). The PHQ‐9: Validity of a brief depression severity measure. Journal of General Internal Medicine, 16(9), 606–613. 10.1046/j.1525-1497.2001.016009606.x 11556941 PMC1495268

[jcv212219-bib-0044] Lundqvist, L. O. , & Lindner, H. (2017). Is the autism‐spectrum quotient a valid measure of traits associated with the autism spectrum? A Rasch validation in adults with and without autism spectrum disorders. Journal of Autism and Developmental Disorders, 47(7), 2080–2091. 10.1007/s10803-017-3128-y 28425021 PMC5487751

[jcv212219-bib-0045] Macdonald, S. J. , & Deacon, L. (2019). Twice upon a time: Examining the effect socio‐economic status has on the experience of dyslexia in the United Kingdom. Dyslexia, 25(1), 3–19. 10.1002/dys.1606 30614597

[jcv212219-bib-0046] Mackintosh, N. (2011). IQ and human intelligence. Oxford University Press.

[jcv212219-bib-0047] Mandell, D. S. , Wiggins, L. D. , Carpenter, L. A. , Daniels, J. , DiGuiseppi, C. , Durkin, M. S. , Giarelli, E. , Morrier, M. J. , Nicholas, J. S. , Pinto‐Martin, J. A. , Shattuck, P. T. , Thomas, K. C. , Yeargin‐Allsopp, M. , & Kirby, R. S. (2009). Racial/ethnic disparities in the identification of children with autism spectrum disorders. American Journal of Public Health, 99(3), 493–498. 10.2105/ajph.2007.131243 19106426 PMC2661453

[jcv212219-bib-0048] Mantzalas, J. , Richdale, A. L. , & Dissanayake, C. (2022). A conceptual model of risk and protective factors for autistic burnout. Autism Research, 15(6), 976–987. 10.1002/aur.2722 35416430

[jcv212219-bib-0049] Meachon, E. J. , Beitz, C. , Zemp, M. , Wilmut, K. , & Alpers, G. W. (2022). The adult developmental coordination disorders/dyspraxia checklist–German: Adapted factor structure for the differentiation of DCD and ADHD. Research in Developmental Disabilities, 126, 104254. 10.1016/j.ridd.2022.104254 35550942

[jcv212219-bib-0050] Muthèn, L. K. , & Muthèn, B. O. (2017). Mplus: Statistical analysis with latent variables. User’s Guide (8th ed.). Muthèn & Muthèn.

[jcv212219-bib-0051] Noordhof, A. , Krueger, R. F. , Ormel, J. , Oldehinkel, A. J. , & Hartman, C. A. (2015). Integrating autism‐related symptoms into the dimensional internalizing and externalizing model of psychopathology. The TRAILS Study. Journal of Abnormal Child Psychology, 43(3), 577–587. 10.1007/s10802-014-9923-4 25099360

[jcv212219-bib-0052] Owen, M. J. , & O'Donovan, M. C. (2017). Schizophrenia and the neurodevelopmental continuum: Evidence from genomics. World Psychiatry, 16(3), 227–235. 10.1002/wps.20440 28941101 PMC5608820

[jcv212219-bib-0053] Pellicano, E. , & den Houting, J. (2022). Annual Research Review: Shifting from ‘normal science’to neurodiversity in autism science. Journal of Child Psychology and Psychiatry, 63(4), 381–396. 10.1111/jcpp.13534 34730840 PMC9298391

[jcv212219-bib-0054] Prolific . (2023). Retrieved from https://researcher‐help.prolific.co/hc/en‐gb/articles/360019236753‐Representative‐samples. accessed on 29 06 2023.

[jcv212219-bib-0055] Pryke‐Hobbes, A. , Davies, J. , Heasman, B. , Livesey, A. , Walker, A. , Pellicano, E. , & Remington, A. (2023). The workplace masking experiences of autistic, non‐autistic neurodivergent and neurotypical adults in the UK. PLoS One, 18(9), e0290001. 10.1371/journal.pone.0290001 37672533 PMC10482295

[jcv212219-bib-0056] Rapoport, J. L. , Giedd, J. N. , & Gogtay, N. (2012). Neurodevelopmental model of schizophrenia: Update 2012. Molecular Psychiatry, 17(12), 1228–1238. 10.1038/mp.2012.23 22488257 PMC3504171

[jcv212219-bib-0057] Reindal, S. M. (2008). A social relational model of disability: A theoretical framework for special needs education? European Journal of Special Needs Education, 23(2), 135–146. 10.1080/08856250801947812

[jcv212219-bib-0058] Robbins, T. W. , Gillan, C. M. , Smith, D. G. , de Wit, S. , & Ersche, K. D. (2012). Neurocognitive endophenotypes of impulsivity and compulsivity: Towards dimensional psychiatry. Trends in Cognitive Sciences, 16(1), 81–91. 10.1016/j.tics.2011.11.009 22155014

[jcv212219-bib-0059] Robertson, A. E. , & Simmons, D. R. (2013). The relationship between sensory sensitivity and autistic traits in the general population. Journal of Autism and Developmental Disorders, 43(4), 775–784. 10.1007/s10803-012-1608-7 22832890

[jcv212219-bib-0060] Rodriguez, A. , Reise, S. P. , & Haviland, M. G. (2016). Evaluating bifactor models: Calculating and interpreting statistical indices. Psychological Methods, 21(2), 137–150. 10.1037/met0000045 26523435

[jcv212219-bib-0061] Rong, Y. , Yang, C. J. , Jin, Y. , & Wang, Y. (2021). Prevalence of attention‐deficit/hyperactivity disorder in individuals with autism spectrum disorder: A meta‐analysis. Research in Autism Spectrum Disorders, 83, 101759. 10.1016/j.rasd.2021.101759

[jcv212219-bib-0062] Roos, J. M. , & Bauldry, S. (2022). Confirmatory factor analysis. SAGE Publications.

[jcv212219-bib-0063] Singer, J. (1999). ‘Why can’t you be normal for once in your life?’ From a ‘problem with no name’ to the emergence of a new category of difference. In M. Corker & S. French (Eds.), Disability discourse. Open University Press.

[jcv212219-bib-0064] Snowling, M. , Dawes, P. , Nash, H. , & Hulme, C. (2012). Validity of a protocol for adult self‐report of dyslexia and related difficulties. Dyslexia, 18(1), 1–15. 10.1002/dys.1432 22271419 PMC3382192

[jcv212219-bib-0065] Spinella, M. (2005). Self‐rated executive function: Development of the executive function index. International Journal of Neuroscience, 115(5), 649–667. 10.1080/00207450590524304 15823930

[jcv212219-bib-0066] Spitzer, R. L. , Kroenke, K. , Williams, J. B. , & Löwe, B. (2006). A brief measure for assessing generalized anxiety disorder: The GAD‐7. Archives of Internal Medicine, 166(10), 1092–1097. 10.1001/archinte.166.10.1092 16717171

[jcv212219-bib-0067] Straub, L. , Bateman, B. T. , Hernandez‐Diaz, S. , York, C. , Lester, B. , Wisner, K. L. , McDougle, C. J. , Pennell, P. B. , Gray, K. J. , Zhu, Y. , Suarez, E. A. , Mogun, H. , & Huybrechts, K. F. (2022). Neurodevelopmental disorders among publicly or privately insured children in the United States. JAMA Psychiatry, 79(3), 232–242. 10.1001/jamapsychiatry.2021.3815 34985527 PMC8733868

[jcv212219-bib-0068] Tennant, R. , Hiller, L. , Fishwick, R. , Platt, S. , Joseph, S. , Weich, S. , Parkinson, J. , Secker, J. , & Stewart‐Brown, S. (2007). The Warwick‐Edinburgh mental well‐being scale (WEMWBS): Development and UK validation. Health and Quality of Life Outcomes, 5(1), 1–13. 10.1186/1477-7525-5-63 18042300 PMC2222612

[jcv212219-bib-0069] Thapar, A. , Cooper, M. , & Rutter, M. (2017). Neurodevelopmental disorders. The Lancet Psychiatry, 4(4), 339–346. 10.1016/s2215-0366(16)30376-5 27979720

[jcv212219-bib-0070] Ward, J. (2019). Individual differences in sensory sensitivity: A synthesizing framework and evidence from normal variation and developmental conditions. Cognitive Neuroscience, 10(3), 139–157. 10.1080/17588928.2018.1557131 30526338

[jcv212219-bib-0071] Zablotsky, B. , Black, L. I. , Maenner, M. J. , Schieve, L. A. , Danielson, M. L. , Bitsko, R. H. , Blumberg, S. J. , Kogan, M. D. , & Boyle, C. A. (2019). Prevalence and trends of developmental disabilities among children in the United States: 2009–2017. Pediatrics, 144(4), e20190811. 10.1542/peds.2019-0811 31558576 PMC7076808

[jcv212219-bib-0072] Zablotsky, B. , Bramlett, M. D. , & Blumberg, S. J. (2020). The co‐occurrence of autism spectrum disorder in children with ADHD. Journal of Attention Disorders, 24(1), 94–103. 10.1177/1087054717713638 28614965 PMC5711626

[jcv212219-bib-0073] Zhu, Y. , Mu, W. , Chirica, M. G. , & Berenbaum, H. (2022). Testing a theory‐driven factor structure of the autism‐spectrum quotient. Autism Research, 15(9), 1710–1718. 10.1002/aur.2763 35665463

