# Supplementary material for: A transdiagnostic approach to neurodiversity in a representative population sample: The N+ 4 model
Source: JCPP Adv. 2024 Feb 1;4(2):e12219. doi: 10.1002/jcv2.12219 (PMC11143952; doi:10.1002/jcv2.12219)
Supplement: Supplementary file 1 — Supplementary Information S1 [file JCV2-4-e12219-s001.docx]

### **Table S1: Descriptive Statistics and correlations for main variables**

| **Variables** | **ASOC** | **AROU** | **AIMA** | **ANAP** | **READ** | **WORD** | **INAT** | **HYPR** | **IMPL** | **SENS** | **MOTT** | **VERT** | **OBSS** | **COMP** | **GROS** | **FINE** | **VISS** | **AURA** | **VISP** |
| --- | --- | --- | --- | --- | --- | --- | --- | --- | --- | --- | --- | --- | --- | --- | --- | --- | --- | --- | --- |
| Social Skills [ASOC] |  |  |  |  |  |  |  |  |  |  |  |  |  |  |  |  |  |  |  |
| Routine [AROU] | .44** |  |  |  |  |  |  |  |  |  |  |  |  |  |  |  |  |  |  |
| Imagination [AIMA] | .34** | .32** |  |  |  |  |  |  |  |  |  |  |  |  |  |  |  |  |  |
| Numbers and Patterns [ANAP] | .05 | .08* | -.06 |  |  |  |  |  |  |  |  |  |  |  |  |  |  |  |  |
| Reading [READ] | .08* | .19** | .24** | -.01 |  |  |  |  |  |  |  |  |  |  |  |  |  |  |  |
| Word-Finding [WORD] | .17** | .18** | .25** | .03 | .48** |  |  |  |  |  |  |  |  |  |  |  |  |  |  |
| Inattention/ Memory Problems [INAT] | .14** | .11** | .25** | .002 | .22* | .39** |  |  |  |  |  |  |  |  |  |  |  |  |  |
| Hyperactivity/ Restlessness [HYPR] | -.19** | -.09** | .004 | .20** | .10** | .13** | .36** |  |  |  |  |  |  |  |  |  |  |  |  |
| Impulsivity/ Emotional Lability [IMPL] | .16** | .27** | .26** | .09** | .21** | .37** | .59** | .49** |  |  |  |  |  |  |  |  |  |  |  |
| Sensory Sensitivities [SENS] | .31** | .29** | .20** | .33** | .28** | .45** | .43** | .32** | .50** |  |  |  |  |  |  |  |  |  |  |
| Motor Tics [MOTT] | .11** | .18** | .15** | .16** | .20** | .26** | .33** | .33** | .39** | .56** |  |  |  |  |  |  |  |  |  |
| Vocal Tics [VERT] | .07* | .12** | .12** | .17** | .22** | .28** | .31** | .34** | .49** | .51** | .56** |  |  |  |  |  |  |  |  |
| Obsessions [OBSS] | .21** | .29** | .16** | .18** | .18** | .26** | .34** | .24** | .49** | .56** | .55** | .55* |  |  |  |  |  |  |  |
| Compulsions [COMP] | .14** | .26** | .13** | .31** | .16** | .21** | .24** | .32** | .40** | .57** | .57** | .56** | .54** |  |  |  |  |  |  |
| Gross Motor Difficulties [GROS] | .24** | .27** | .24** | .06 | .24** | .41** | .46** | .14** | .45** | .56** | .39** | .37** | .42** | .32** |  |  |  |  |  |
| Fine Motor Difficulties [FINE] | .16** | .14** | .22** | .08** | .38** | .36** | .42** | .16** | .36** | .46** | .33** | .32** | .33** | .26** | .51** |  |  |  |  |
| Heightened Visual Sensitivity and Discomfort [VISS] | .23** | .23** | .11** | .19** | .19** | .36** | .31** | .16** | .39** | .67** | .39** | .38** | .41** | .39** | .50** | .40** |  |  |  |
| Aura-like Hallucinatory Experience [AURA] | .12** | .12** | .03 | .24** | .15** | .26** | .27** | .21** | .32** | .50** | .40** | .40** | .38** | .39** | .32** | .33** | .64** |  |  |
| Distorted Visual Perception [VISP] | .09** | .15** | .02 | .28** | .17** | .24** | .28** | .27** | .35** | .58** | .44** | .45** | .43** | .47** | .36** | .34** | .69** | .75** |  |
| **Number of Participants [N]** | 983 | 983 | 983 | 983 | 989 | 989 | 988 | 988 | 988 | 989 | 989 | 989 | 989 | 989 | 989 | 989 | 981 | 981 | 981 |
| **Mean score on scale [M]** | 11.98 | 7.46 | 8.19 | 11.42 | 2.84 | 3.90 | 10.17 | 10.20 | 9.13 | 40.50 | 1.22 | .94 | 1.88 | 1.05 | 24.41 | 14.70 | 35.29 | 10.34 | 7.73 |
| **Standard Deviation [SD]** | 3.46 | 1.84 | 2.25 | 3.28 | 2.16 | 2.07 | 6.55 | 6.36 | 6.35 | 19.65 | 1.80 | 1.50 | 2.35 | 1.45 | 8.03 | 4.98 | 26.02 | 13.81 | 9.67 |
| **Cronbach’s Alpha [α]** | .81 | .59 | .61 | .76 | .66 | .63 | .88 | .87 | .90 | .92 | .77 | .70 | .87 | .64 | .89 | .86 | .95 | .92 | .89 |

**p < 0.01 (2-tailed) *p < 0.05 (2-tailed)

**Power analysis: Sample Size Rationale using Monte Carlo Simulations**

Using Monte Carlo simulations for the most complex of the models we intended to test (i.e., 7 condition factors and 1 bifactor variable) and assuming moderate loadings of the 22 indicators on the condition factors (.35) and weak-to-moderate loadings on the bifactor (.30), we calculated that 500 participants would provide sufficient power (.86-.99) to detect significant loadings on all factors. 1000 participants therefore enabled us to test competing models in one half of the data and cross-validate our best-fitting final model within the second half of the sample (randomly selected).

**Table S2 Model Fit Indices for Structural Equation Models.**

|  | **Description** | $\boldsymbol{\chi}^{\boldsymbol{2}}$ | ***df*** | **CFI** | **TLI** | **RMSEA** | **AIC** | **BIC** |
| --- | --- | --- | --- | --- | --- | --- | --- | --- |
| 1 | Demographic correlates of Neurodiversity | 969.39 | 171 | 0.89 | 0.85 | 0.07 | 114310.06 | 114819.94 |
| 1A | Demographic correlates of Neurodiversity with modifications. | 670.07 | 163 | 0.93 | 0.90 | 0.06 | 113992.87 | 114541.98 |
| 2 | Executive Function and Non-Verbal Ability Model | 1028.08 | 233 | 0.92 | 0.88 | 0.06 | 137685.09 | 138528.36 |
| 2A | Executive Function and Non-Verbal Ability Model with modifications. | 828.65 | 227 | 0.94 | 0.91 | 0.05 | 137476.81 | 138349.50 |
| 3 | Wellbeing and Mental Health Model | 842.27 | 219 | 0.94 | 0.91 | 0.05 | 134341.98 | 135116.61 |

*Note.* 1A. Modification indices suggested direct effects between gender and Heightened Visual Sensitivity (CHI-II), Fine Motor Skills (ADC-R), Impulsivity (CAARS), Vocal Tics (MOVES), and Word Finding Difficulties (ARQ). Modification indices also suggested direct effects between age and Preference for Routine (AQ), Inattention (CAARS), and Reading Difficulties (ARQ). 2A. Modification indices suggested direct effects between Strategic Planning (EFI) and Inattention (CAARS), Hyperactivity (CAARS) and Preference for Numbers and Patterns (AQ). There were direct effects between Impulse Control (EFI) and Vocal Tics (MOVES) and Sensory Sensitivity (GSQ) and between Organisation (EFI) and Inattention (CAARS).

### **Table S3 Robust Maximum Likelihood Estimates for Bifactor Model Loadings.**

|  | N Factor | | | A Factor | | | B Factor | | | C Factor | | | D Factor | | |
| --- | --- | --- | --- | --- | --- | --- | --- | --- | --- | --- | --- | --- | --- | --- | --- |
|  | Est. | SE | *p* | Est. | SE | *p* | Est. | SE | *p* | Est. | SE | *p* | Est. | SE | *p* |
| ASOC | .27 | .04 | *** | .65 | .04 | *** |  |  |  |  |  |  |  |  |  |
| AROU | .34 | .03 | *** | .54 | .04 | *** |  |  |  |  |  |  |  |  |  |
| AIMA | .25 | .04 | *** | .40 | .04 | *** |  |  |  |  |  |  |  |  |  |
| ANAP | .28 | .03 | *** |  |  |  | -.28 | .04 | *** |  |  |  |  |  |  |
| READ | .31 | .03 | *** |  |  |  | .18 | .05 | *** |  |  |  |  |  |  |
| WORD | .45 | .03 | *** |  |  |  | .35 | .04 | *** |  |  |  |  |  |  |
| INAT | .50 | .03 | *** |  |  |  | .37 | .37 | *** | .43 | .04 | *** |  |  |  |
| HYPR | .39 | .03 | *** | -.42 | .03 | *** |  |  |  | .46 | .04 | *** |  |  |  |
| IMPL | .63 | .03 | *** |  |  |  |  |  |  | .58 | .04 | *** |  |  |  |
| MOTT | .72 | .02 | *** | -.12 | .03 | *** |  |  |  |  |  |  |  |  |  |
| VERT | .70 | .02 | *** |  |  |  |  |  |  |  |  |  |  |  |  |
| OBSS | .72 | .02 | *** |  |  |  |  |  |  |  |  |  |  |  |  |
| COMP | .74 | .02 | *** |  |  |  | -.30 | .04 | *** |  |  |  |  |  |  |
| GROS | .62 | .03 | *** |  |  |  | .42 | .04 | *** |  |  |  |  |  |  |
| FINE | .52 | .03 | *** |  |  |  | .40 | .04 | *** |  |  |  |  |  |  |
| VISS | .62 | .03 | *** |  |  |  |  |  |  |  |  |  | .45 | .03 | *** |
| AURA | .56 | .03 | *** |  |  |  |  |  |  |  |  |  | .64 | .03 | *** |
| VISP | .63 | .03 | *** |  |  |  |  |  |  |  |  |  | .64 | .03 | *** |
| SENS | .82 | .02 | *** |  |  |  |  |  |  |  |  |  |  |  |  |

*Note.* ***p<.001. **p<.01. *p<.05. SENS = GSQ Sensory Sensitivities. MOTT = MOVES Motor Tics. VERT = MOVES Verbal Tics. OBSS = MOVES Obsessions. COMP = MOVES Compulsions. ASOC = AQ Social Skills Difficulties. AROU = AQ Preference for Routines. AIMA = AQ Imagination. ANAP = AQ Numbers and Patterns. INAT = CAARS Inattentiveness. HYPR = CAARS Hyperactivity. IMPL = CAARS Impulsivity. VISS = CHI Heightened Visual Sensitivity. AURA = CHI Aura-Like Hallucinatory Experiences. VISP = CHI Distorted Visual Perception. READ = ARQ Reading Difficulties. WORD = ARQ Word Finding Difficulties. GROS = ADCDQ Gross Motor Skills. FINE = ADCDQ Fine Motor Skills.
